# Talking together in rural palliative care: a qualitative study of interprofessional collaboration in Norway

**DOI:** 10.1186/s12913-022-07713-z

**Published:** 2022-03-07

**Authors:** May-Lill Johansen, Bente Ervik

**Affiliations:** 1 Research Unit for General Practice, Department of Community Medicine, Faculty of Health Sciences, UiT The Arctic University of Norway, N-9037 Tromsø, Norway; 2 Department of Oncology, University Hospital of Northern Norway, Tromsø, Norway

**Keywords:** Palliative home care, End-of-life care, General practice, Interprofessional collaboration, Transitions of care, Norway

## Abstract

**Background:**

Caring for people with palliative care needs in their homes requires close collaboration within and between primary and hospital care. However, such close collaboration is often lacking. Transitions of care are potentially unsafe and distressing points in a patient trajectory. Few studies have explored the experiences of healthcare professionals in the community who receive patients from hospital care and provide them with palliative care at home.

**Objective:**

To explore how rural health professionals experience local and regional collaboration on patients in need of palliative care.

**Methods:**

This was a qualitative focus group and interview study in rural Northern Norway involving 52 primary care health professionals including district nurses, general practitioners, oncology nurses, physiotherapists, and occupational therapists. Five uni-professional focus group discussions were followed by five interprofessional discussions and six individual interviews. Transcripts were analysed thematically.

**Results:**

“Talking together” was perceived as the optimal form of collaboration, both within primary care and with specialists. Nurses and GPs had similar perceptions of their worst-case scenario in primary palliative care: the sudden arrival after working hours of a sick patient about whom they lacked information. These situations could be the result of a short notice transfer from secondary care or an emergency presentation after a crisis in patient management locally, the latter often resulting in a hospital admission. Participants missed timely and detailed discharge letters and in complex cases a telephone call or conference. Locally, co-location was perceived as advantageous for crucial communication, mutual support, and knowledge about each other’s competencies and work schedule. Because local health professionals belonged to different units within the primary health care organisation, in some places they had limited knowledge about each other’s roles and skill sets.

**Conclusions:**

Lack of communication, both locally and between specialist and primary care, was a key factor in the worst-case patient scenarios for GPs and nurses working in primary palliative care in rural Northern Norway. Co-location of primary care professionals promoted local collaboration and should be encouraged. Hospital discharge planning should involve the receiving primary care professionals.

## Background

Palliative care at the end of life often involves complex issues and challenges for patients, families, and health services [1]. In many countries, primary care is increasingly assuming responsibility for patients with palliative needs. However, the degree of collaboration between professionals in primary care can vary from well-organised teamwork to fragmented services [2]. In rural areas, due to the limited number of members of each health profession, ad hoc teams may form as required, but not always [3]. It is well recognised that patients with complex conditions, such as those requiring palliative care, benefit from collaboration in health care, while fragmentation of health care services strongly affects these highly vulnerable patient groups [4].

Caring for patients at the end of life in their homes ideally requires close collaboration between primary and secondary health care [5], including professionals from several disciplines. Many patients want to spend their last days at home or as close to home as possible; however, their medical condition might need assessment by a specialist in palliative care including referral to hospital care for some days [6]. Transitions of care between hospital and home and vice versa are potentially unsafe and distressing points in a patient trajectory [7, 8]. People in need of palliative care are at increased risk of unsafe care transitions, particularly in out-of-hours contexts [9]. High quality home care requires clinicians to establish relationships with and mediate between different services to optimise care of patients and carers [10]. Despite advances in technology, communication challenges persist, sometimes with serious consequences for clinical decision making [11]. There is a lack of studies exploring the experiences of healthcare professionals in the community who receive patients from specialist hospital care and provide them with palliative care at home [12].

### Study setting

Health services in Norway are predominantly public and free of charge. The municipalities are responsible for primary health care, including primary palliative care, while specialist health care is provided by the four health regions and only following referral from primary care. Public district nurses (DNs) and small general practices of mainly self-employed general practitioners (GPs) make up the backbone of primary care. Northern Norway, one of the four health regions in Norway, mainly consists of vast rural areas with a scattered population. At the time of the study, 52 of the 87 municipalities in Northern Norway employed nurses with a postgraduate diploma in oncology nursing, including palliative care, hereafter called oncology nurses (ONs). Most municipalities had physiotherapists (PTs) and a few had an occupational therapist (OT). Often self-employed, GPs have more autonomy in municipal primary care organization than the other health professionals. Together, these primary care professionals can make up a local municipal palliative team, in an ad hoc manner as required. However, municipalities vary in their organization of primary palliative care.

In Norway, palliative care is integrated in the public health services. Specialist palliative care centres, located in the regional hospitals, have at least one palliative care physician and one ON. These palliative care specialists are consulted within the hospital and by primary care clinicians (GPs and ONs), who can also refer patients to them. However, from the most remote communities it can take up to four hours to drive to the nearest hospital. Due to the long distances and sparse population, there is no hospice in Northern Norway. Instead, end-of-life care is provided locally; in the patient’s home, in nursing homes, or in rural medical centres.

### Aim

This study aimed to explore how rural health professionals in Northern Norway experience collaboration regarding palliative care patients, both locally and with hospital-based specialists, including perceived facilitators and barriers to optimal collaboration and the consequences of non-optimal collaboration.

## Methods

Data were gathered through focus groups and individual interviews in 2015–2016. The first five focus groups were held with nurses and GPs separately to discuss their experiences of interdisciplinary work in palliative care without the other profession being present. The recruitment process for the uni-professional groups was both purposive (gathering professionals with varied backgrounds from municipalities of varied sizes in different geographical locations) and pragmatic (recruiting professionals who were going to be present at an educational event, to avoid extra travelling). We had two groups of GPs and three groups of nurses, comprising both DNs and ONs. Each focus group had 3–8 participants. The first group of GPs was recruited by e-mailing purposively selected participants on a continuing medical education course. The second GP group was created by snowballing from the first one. Nurses were recruited by e-mailing purposively selected participants on a palliative course and at two palliative care network meetings. The focus group discussions (FGDs) were held in meeting rooms at the same premises as the educational events. Geographically, the GPs and nurses came from 19 different municipalities with a median population of 3028 inhabitants.

To obtain more detailed and contextual knowledge on local collaboration, six rural municipalities were purposively selected for their variation in size, geography, and organisation of palliative care. BE asked the ON in each municipality to organise a group of professionals that would normally work together in palliative care. The participants were ONs, GPs, DNs, PTs, and OTs. In one municipality, six different professionals were interviewed individually at their health centre. Here, we wanted to gain a detailed picture of the role of each professional and their views on collaboration. We then conducted FGDs in the five other municipalities selected, each with 3–7 professionals, aiming for discussion and interaction between the participants [13, 14]. See Tables 1 and 2 for an overview.

**Table 1:** Participants in the study

Profession	*N*	Age range (years)	Professional experience (median years)	Gender
District nurses	15	26–61	11 (2–35)	All women
Oncology nurses	15	35–60	14 (9–37)	All women
General practitioners	17	27–68	11 (0–34)	6 men, 11 women
Physiotherapists Occupational therapists	5	27–55	12 (0–26)	1 man, 4 women
Total	52

**Table 2:** Focus groups and interviews

Chronological order of data collection	Profession	Groups	Persons	Municipalities represented
First	GP FGDs	2	8	5
Second	Nurse FGDs	3	16	14
Third b)	Team FGDs	5	22	5
	Total FGDs	10	46	24
Third a)	Interviews		6	1
	Total		52	25

The interview guides consisted of a brief topic guide jointly developed by the authors [15]. We encouraged participants to share and discuss authentic personal experiences. The FGDs, lasting around 90 min, were mediated by BE and MLJ. BE is an ON by background while MLJ is an academic GP. Both have experience of qualitative methods from their PhDs and postdoctoral research. The individual interviews were conducted by medical student Birgit Brøndbo as part of her master’s thesis, supervised by MLJ. These interviews lasted around one hour. The authors had a running dialogue about preliminary findings during data collection and fed emerging questions into subsequent FGDs. The ten FGDs and six interviews were digitally recorded and transcribed verbatim. Under Norwegian legislation, this project did not need ethics approval.

We conducted an inductive thematic analysis within a realist paradigm, and were interested in both semantic and latent content [16]. Thematic analysis is well suited to identify patterns in a large dataset such as ours. We coded the transcripts independently, looking for recurrent topics, patterns and “critical incidents that can illuminate the research questions” [17]. Potential overarching themes and subthemes were discussed between the researchers, revised, and refined in an iterative process. All coded text on the final themes and subthemes was then extracted, collated, and condensed. We aimed to illustrate subthemes with a rich selection of quotes. See Table 3 for a thematic map.

**Table 3:** Thematic map

RQ1	Final theme	Facilitators	Barriers
Optimal collaboration	“Talking together”	Co-location Ad hoc conversations Scheduled meetings	Not knowing each other E-messages replace talking Lack of meetings
**RQ2**	**Final theme**	**Consequences**	
Non-optimal collaboration	Care transitions without “talking together”	Unsafe care Sub-optimal treatment Lack of shared goals	

*RQ* research question

To assure trustworthiness of our results, we used several strategies [18]. For dependability, we kept an audit trail of the decisions during the analytic process. For transferability, we described characteristics of the research region, the municipalities involved, and our participants. For credibility and confirmability, we discussed our emergent findings with resource persons in the field, such as GPs, ONs, and fellow researchers.

## Results

“Talking together” was perceived as the optimal form of collaboration, locally and regionally. Co-location of services, ad hoc conversations, and scheduled meetings facilitated “talking together”, while lack of meetings and knowledge of each other’s work obstructed optimal collaboration. Not “talking together” was seen as a threat to safe, collaborative patient care and shared treatment goals, as elaborated below.

### Transitions of care: worst case scenarios of not talking together

During our FGDs, it became clear that there was one situation that was equally feared by doctors and nurses alike but from different perspectives. This worst-case scenario involved being the nurse on a late shift or being the GP on call after hours.

From the nurses’ perspective, the scenario was about assuming responsibility for a patient at the end of life without a plan for discharge to primary palliative care and then having to contact the GP on call, worrying that the GP would not know the patient or which medication to prescribe. This scenario could arise from a sudden patient transfer from specialist care without primary care having been consulted about the patient’s situation.

ON T5: *Two weeks ago, we had a (…) seriously ill COPD patient admitted to the hospital (…), and then I wasn’t told until the handover that the patient was going home today. (…) it was in a letter from the doctor at the hospital saying that he had no more treatment to offer (…). And then I really expected them to maybe have prescribed some painkillers … But there was nothing about that in the papers. And the patient got so bad during the afternoon and evening that I had to call the emergency GP here, who fortunately came to monitor the patient. And the patient died the next morning … Well, then you’d like to have had a bit more clarification from the specialist health services.*

GPs found their role as coordinators of care difficult to fulfil when patients’ previous palliative treatment had been in the hands of hospital medical specialists. In the uni-professional focus groups, GPs talked about how they could lose track of their patients. As long as the patient’s symptoms were well controlled and the family caregivers coped with the situation, the lack of involvement of the GP did not have any major consequences. However, when a crisis in home care emerged, the patient’s GP, or the doctor on call after hours, had to find a solution. Such an after-hours crisis was the GPs’ worst-case scenario. With no knowledge of the patient’s situation and preferences, hospital admission was often difficult to avoid, with the accompanying stress of travelling for patient and family. The GPs also commented on their responsibility to prevent these situations by talking to their patients about their wishes for their end of life and documenting this in a transparent way.

GP D2: *Maybe a patient has had plenty of contact with the specialist health services, and then it kind of breaks down, and when it breaks down, well, that’s it, so then we just say, what do we do now? And there will probably be some unnecessary hospital admissions, because then they end up in the emergency clinic and then you don’t know … but is this really a terminal process, should the patient go to the rural medical centre instead or what, and then that doctor doesn’t know about that, it can be difficult to have the necessary discussion about this out-of-hours, and then perhaps instead the patient will be sent to the main hospital.*

The most common gap in communication around transitions of care was the lack of timely discharge letters. This was a problem for GPs, ONs, and staff in nursing homes with palliative beds. When patients were transferred to them from hospitals and the primary care staff had not received any discharge letters or reports, it was difficult to follow the previous care plan and to establish trust with the patient and family in the care transitions. Even if they had received a discharge letter, there was often insufficient information in the letter about patients with complex conditions.

Receiving timely and adequate information and talking together about a care plan for patients with complex palliative needs before discharge was regarded as very important. Many participants would in these cases have liked to receive a phone call from the hospital or to take part in a planned discharge meeting, ideally held as a video conference. One GP explained that talking with a specialist about a particular patient meant negotiating between different forms of expertise.

GP I1: *Mostly, I find that when I call the hospital (…), we can have a dialogue around the patient and a discussion, so we reach a common solution (…). We have different expertise. I’m a specialist in general practice, I might be talking to a cancer specialist. (…) And then I’m the one who knows the patient best. And then the oncologist knows his subject best, you see. So, then we can meet halfway and say, hey, this works, or this doesn’t work. (…) To supplement a referral (…) you should maybe often have a telephone call.*

### Co-location and improvised consultancy: “Could I have a couple of words with you?”

Health professionals who worked together in community palliative care talked about co-location as highly beneficial to their cooperation and those who were not co-located commented that they missed it. The closer the offices to each other, the easier it was to pop into each other’s office and talk about shared concerns such as changes observed in a shared patient. For ONs, it was good to be located close to the GPs. The ON could then easily visit the doctor for a discussion or to obtain a prescription. Conversely, doctors could easily update themselves on a patient’s palliative care trajectory when the ON, the PT, or the OT knocked at their door and asked: “Could I have a couple of words with you?” When working so close to each other, no issue ever got ‘old’; if clinicians were wondering about something, they could bring it up immediately. GPs who had a co-located ON were grateful for this. They could talk in the corridor and meet in the canteen. Contact was easy and informal, which they regarded as important. The co-located ONs talked about how they appreciated the support and opportunities for debriefing sessions offered by doctors.

PT T3:* (…) because you’ve actually physically met the other person and you talk just the two of you and close the door and sometimes you bring up serious things that only take two minutes, but those two minutes are very serious, you get more out of it then than sending an email and getting … because it will never be the same.*

On the other hand, not being co-located had disadvantages. Doctors felt that they missed being easily contacted by the ONs about shared patients. ONs whose offices were far away from the GP practice noted the lack of opportunities to discuss care informally. Distant locations could give clinicians a feeling of not belonging together in a primary care organisation. Thus, for some DNs and ONs, contacting secondary care for advice instead of the patients’ regular GP often felt like the easier option.

Some municipalities took part in a network that had regular interprofessional video conferences with the regional oncology department. During these conferences, health professionals would discuss patients’ clinical situation and make joint decisions, saving patients and families from travelling to the hospital. On other occasions, GPs would call the hospital for advice during a consultation and turn up the volume of the palliative care specialist to enable the patient to take part in the discussion. The opportunity for such improvised telephone conferences with specialists was highly appreciated.

### Lack of meetings and knowledge of each other

In recent years, e-messaging through the secure national electronic health platform had become a common form of communication between local primary care professionals. These messages often replaced phone calls or meetings. In this way, ongoing patient care was less disturbed by phone calls and e-messages were copied into the electronic patient record as a safer transfer of information. The e-messages worked well for asking simple questions, one at a time, responding to these, and handing over short items of information.

However, participants agreed that e-messages could not replace personal contact. Some GPs missed meetings with the DNs to discuss patients. They also missed DNs accompanying shared patients to consultations. The doctors understood that the DNs were busy but claimed that the quality and safety of consultations could be jeopardised if certain patients came unaccompanied. DNs also missed the meetings that they used to have with GPs, before e-messages were introduced.

Participants noted that a lack of meetings could mean that clinicians did not have common goals for their palliative care, and thus no team approach. They perceived a need to physically meet to routinely share information, assessments, and evaluations of patients’ situations and discuss how this was related to their own work.

PT T2: *If you agree on what to do and so on, you can often provide better care. We know about each other’s goals and what we think and so on … Like if my goal is to get the person out of bed and walking again and then she thinks that the person will only live for another two weeks, you know.*

Some GPs talked about how easy they found cooperation within their own profession because of the knowledge they had of each other’s competencies and work schedule. This was compared to their knowledge about the ONs, which was often limited. GPs did not always know about the health services of the ONs, even within a relatively small town. Moreover, although different health professionals often had their offices in the same building, they were not close enough to each other to meet by chance.

## Discussion

The overall aim of our project was to gain knowledge about how to improve primary palliative care through better collaboration. Other papers from the project are exploring collaboration between ONs and GPs [19], equal access to palliative care [20], and the meaning of home in end-of-life care.

The experiences of our participants suggest that formal and informal opportunities for talking together are a matter of providing co-ordinated collaborative patient care, including patient safety. Although recent Norwegian policy documents indicate a need for palliative care pathways and a focus on improving fragmented services and uncoordinated transitions of care [21, 22], these shortcomings are rarely discussed as safety issues. There is evidence connecting patient safety to well-functioning collaboration amongst health professionals, as communication failures account for the majority of adverse events in patients [23]. Our participants perceived delayed discharge letters and lack of direct contact between hospitals and primary care as a safety risk. GPs could feel that they had lost track of their patients in need of palliative care. In another Norwegian study [24], GPs missed being contacted by hospital colleagues to discuss patient treatment and suggested that such contact would benefit patients and family caregivers and increase patient safety.

These experiences are in line with numerous studies showing that direct communication between hospitals and primary care doctors occurred infrequently [25, 26], discharge summaries were delayed [25–28], and, like referral letters [27], often lacked important information [25–28]. A study from rural Australia [29] indicated that patients had an assumed sense of safety mediated by a trusting and enduring GP-patient relationship. However, during transitions between primary and secondary care, patients and family caregivers were often responsible for coordinating information between healthcare providers, which was impossible for the most frail and vulnerable patients. Also in Spain, patients were expected to transfer information without being empowered to understand and act on it, possibly leading to misinformation, medical errors, and patient harm [30]. Being discharged on a Friday afternoon was risky, particularly when reports, prescriptions, medications, and essential equipment for home care were lacking [20, 24, 27].

Limitations in the palliative care experience of the GP on call out of hours was another fear of nurses on the late shift. In a previous paper from this study, we found that there were unfortunate variations in palliative knowledge and skills among GPs [19]. This could be because each GP has few patients needing palliative care and limited experience could make it difficult to maintain knowledge and skills [31]. In addition, some patients continue to be followed up by specialist palliative care even after discharge, especially if they live close to a hospital [32]. In our study, lack of GP involvement in a patient’s palliative care until home care broke down could lead to emergency admissions, as also reported from England [33].

What could have prevented our participants’ worst-case scenario? The ideal situation would have been an existing palliative care plan [34], shared between the patient, family, and professionals from primary and secondary care, who would all meet, preferably in the patient’s home, and talk together. Such plans are increasingly being introduced in Norway [22, 35]. Both in Norway and internationally, one key question is where end-of-life discussions should be initiated, in primary or secondary care [36].

Co-location of primary health care services is recommended in a Norwegian white paper on primary care [37]. Even if co-location alone does not make a team, our co-located participants highly recommended being able to work within walking and talking distance of each other. The frequent exchange of “a couple of words” seemed to be the glue in ensuring local cooperation. This resonates well with reviews on interprofessional collaboration in primary care, where the opportunity for frequent informal communication was regarded as the most important factor in effective collaboration [38, 39]. These brief exchanges were necessary for creating shared knowledge, shared goals, and shared clinical decision making. “Favourable physical space configuration and ‘having frequent brief time in common’ were key facilitators” [38]. An extreme example of faulty cooperation and inappropriate organisation was when participants from the same small town reported not knowing about each other’s health services. Also, at other locations, lack of knowledge of each other’s work situation and competence was a barrier to interprofessional cooperation.

A large European study [40] found that from the perspective of palliative care patients and family caregivers, it was essential to receive individualised care and have easy access to help in trusted relationships with a small group of health care professionals. Patients treated by multidisciplinary teams often missed relational continuity with their GPs [40]. A regional patient pathway project in Norway, the Orkdal Model [41], attempts to reconnect discharged palliative cancer patients with their GPs, through palliative care courses, joint home visits, and better supervision for GPs from specialist palliative care, as also recommended in the UK Gold Standards Framework.

For our GP participants, conferring with specialists meant negotiating between different types of knowledge, and possibly different epistemologies. While the medical specialists had advanced assessment and treatment knowledge, the GPs often had personal and contextual knowledge about their patients, which could be important for planning their health care. GPs in other Norwegian studies [24, 42] have described being trusted translators for their patients during phases of hospital-led treatment and palliative care. GPs said that they generally knew their patients well [24, 42] and that patients would benefit if hospital doctors more often consulted with GPs before starting new treatment [24]. A large UK study similarly found that *knowledge sharing* and *knowledge brokering* could help to mitigate the system complexity of care transitions and support collaboration [43].

### Strengths and limitations

We conducted a broad, mainly purposive recruitment of 52 health professionals from rural parts of Northern Norway and obtained rich data from ten FGDs and six interviews. However, we did not include hospital specialists, patients, or family caregivers, which would need further studies. The interprofessional FGDs were not planned as interventions; however, in some groups, solutions to the problems described by the participants of insufficient talking together were worked out in real time.

We are researchers from two disciplines: BE is an oncology nurse and head of the Regional Advisory Unit for Palliative Care, while MLJ is an academic GP. These positions gave us valuable insights, access, and credibility, and although we used an interview schedule, they also influenced our roles as moderators, the topics participants chose to talk about and the way the discussions were interpreted.

Although the study concerned palliative care and was undertaken in the rural North, we believe that our findings may be transferable to other primary care settings in countries with similar services.

## Conclusions

Lack of communication, both locally between primary care professionals and between those in primary and secondary care, was a key factor in the worst-case patient scenarios for GPs, DNs, and ONs working in primary palliative care in Northern Norway. Discharge planning for patients in need of palliative care should involve the receiving primary care professionals, and specialist clinicians should involve GPs earlier to enable them to maintain contact with these patients.

Although electronic communication between health professionals was convenient and suitable for minor queries, it could not replace talking together. This suggests that co-location of primary care professionals to promote local collaboration should be encouraged.

## Data Availability

Data collected for this study will not be made publicly available because the Norwegian Centre for Research Data requires data to be stored on a password-protected file on a university server to ensure confidentiality and privacy. Access to the data is only permitted to the investigators for a designated period.

## Abbreviations

GP: General practitioner
ON: Oncology nurse with an advanced qualification in palliative care
DN: District nurse (home care nurse, community nurse)
PT: Physiotherapist
OT: Occupational therapist
I1: Individual interview number 1
T2, T3, T5: Focus groups numbers 2, 3 and 5 with local teams
D2: Focus group number 2 with doctors
